# Perspective-Taking and Perspective-Sharing in Pediatric Education: Exploring Connections Between Strategies of Medical Students and Patients’ Caregivers

**DOI:** 10.5334/pme.412

**Published:** 2023-10-06

**Authors:** M. C. L. Eijkelboom, R. A. M. de Kleijn, L. Baten, J. Frenkel, M. F. van der Schaaf

**Affiliations:** 1 Faculty of Medicine, Utrecht University, the Netherlands; 2 Department of Pediatrics, University Medical Center Utrecht, the Netherlands; 3 Utrecht Center for Research and Development of Health Professions Education, University Medical Center Utrecht, the Netherlands; 4 Faculty of Medicine, Utrecht University, the Netherlands at the time she contributed to the research

## Abstract

**Introduction:**

In pediatric education, caregivers are increasingly involved to share their perspective. Yet, an in-depth understanding of the perspective-taking process between medical students and caregivers is lacking. This study explored: 1) Which strategies do medical students use to take a caregiver’s perspective and which facilitators and constraints do they perceive? 2) Which strategies do caregivers use to share their perspective with students? and 3) How do students’ perspective-taking strategies relate to caregivers’ perspective-sharing strategies?

**Methods:**

In an online lesson: two caregivers of pediatric patients, shared their story with 27 fourth-year Dutch medical students. After the session, students undertook an assignment where they individually reflected on how they took perspective. Students’ reflections were collected via audio recordings. Caregivers were individually interviewed. Data were analyzed through thematic and cross-case analysis.

**Results:**

Students used eight perspective-taking strategies, in various combinations. Students used inferential strategies, where they made inferences from available information, and cultivating strategies, where they attempted to elicit more information about the caregiver. Students perceived individual-, contextual- and caregiver-related facilitators and constraints for taking perspective. Caregivers shared their perspective by adopting multiple strategies to share their story and create a trusting learning environment. We visualized connections between students’ perspective-taking strategies, facilitators/constraints, and caregivers’ perspective-sharing strategies.

**Discussion:**

By combining data from both perspective-takers (students) and perspective-sharers (caregivers), this study provides a foundation for future research to study perspective-taking between students and patients in an educational context. On a practical level, our findings provide tools for students, patients, and educators to enhance perspective-taking processes.

## Introduction

“It is as important to know what kind of man has the disease, as it is to know what kind of disease has the man.” –William Osler [1]

An important goal of medical education is to foster person-centeredness [2]. Understanding a patient’s concerns, experiences, and preferences is essential to provide care adjusted to their needs [3]. However, valuable opportunities for patients to share their perspectives with individual students are being diminished by the trend to move clinical practice from the bedside to desktop computers and the shortening of inpatient admissions. Therefore, it is ever more important for medical education to introduce students to various patient perspectives and to support them in gaining an understanding of those perspectives [4,5,6]. Increasingly patients are involved in medical education and new initiatives are developed in which students learn about patient perspectives from patients themselves. For instance by interviewing patients with chronic conditions [7], following a patient’s daily regime [8], home visiting families of children with special needs [9], or filming patients with serious chronic illness in their daily lives [10]. However, reviews on patient involvement have demonstrated that little is known on *how* students learn from patients [11,12,13]. As such, an in-depth understanding of the perspective-taking process between students and patients during educational encounters is lacking. Insight into how students take perspective and how patients share their perspective, can inform the development of these new initiatives and ultimately support students in becoming person-centered doctors.

So far, research in health-care contexts focusses on self-reported motivation and engagement in perspective-taking, using questionnaires like the Jefferson Scale of Empathy [14] or the Interpersonal Reactivity Index [15], and on outcomes of perspective-taking such as accuracy or patient satisfaction [16,17,18]. Yet, it is relatively unknown what happens between motivation and outcome: what do students do when they engage in perspective-taking and how do patients-as-teachers share their perspective? Therefore, in this study we aim to better understand the perspective-taking process between students and patients by using social psychology literature, which has studied the perspective-taking process extensively and developed frameworks that might be applicable to the medical education context.

In this study, we conceptualize perspective-taking as the process through which a perceiver, in this study students, discerns the thoughts, feelings and motivations of a perspective-sharer, in this study patients [19]. Studies in social psychology show that multiple strategies can be adopted to understand another’s perspective [20,21,22,23,24]. Batson *et al*. distinguished between two strategies: imagining how another perceives a situation (imagine-other strategy) and imagining how you would perceive a situation yourself (imagine-self strategy) [22]. Outcome-based studies, where participants are instructed to adopt either one of these strategies, show that the imagining-other strategy leads to higher accuracy and less personal distress, compared to the imagining-self strategy [17,22,25]. Several experimental studies, including studies in the medical context, are built on this dichotomy of perspective-taking strategies [17,18,22]. Yet, exploratory studies which are focused on real-life experiences show that people adopt a greater variety of strategies [20,21,23,26]. Moreover, people do not use one single strategy, rather they use combinations of strategies to take perspective [20,21,23,26]. Accordingly, and based on empirical data, Gehlbach *et al*. developed a taxonomy of perspective-taking strategies. This taxonomy consisted of 12 different strategies, which are organized in two broad categories: strategies that facilitate information gathering to learn more about a perspective-sharer (information cultivation strategies) and strategies in which people make inferences from available information (inferential strategies) [20]. For example, Batson’s imagine-other and imagine-self strategies would fit in this taxonomy within the category of inferential strategies. In this study, we used Gelbach’s taxonomy of perspective-taking strategies as a base to understand the perspective-taking process between students and patients [20].

The ability to take another’s perspective is not something which people possess to a certain degree at a certain point in time. Rather, it varies depending on the context and the perspective-sharer [24]. Perspective-taking is inherently an interpersonal process: at least two people are involved in the process, namely the perceiver and the sharer [27]. The relationship between these people [28], their interaction [29,30], characteristics of the perspective-sharer [31] and characteristics of the perspective-taker [32] all shape the perspective-taking process. For instance, where being different from the sharer constrains perspective-taking, familiarity with the perspective-sharer or having a similar past experience increases accuracy [28,33]. Moreover, the context can provide facilitators or constraints for perspective-taking. For instance, someone is better able to take perspective in an environment with few distractors, no time-pressure, and with various possibilities for interaction [34]. In sum, perspective-taking is a process that unfolds over time and depends on the people involved and the context in which the process takes place.

Since perspective-taking is an interpersonal process, the interaction between the perspective-taker and perspective-sharer shapes the process [29,30]. Patients are increasingly invited to share their perspective in medical education. However, how patients share their story in an educational context has not often been researched. Hence, little is known about the strategies patients use to share their perspectives with medical students. Let alone, how patients’ perspective-sharing strategies relate to students’ strategies to take perspective.

This study was performed in the context of a pediatric course. In pediatric care, communication with patients’ caregivers (parents, family members) and understanding their perspectives is an important element of family-centered care [35]. As such, besides patients, their caregivers are also increasingly involved in medical education [9,36,37,38]. Therefore, in this study we explore the perspective-taking process between caregivers-as-teachers and medical students.

In sum, to guide medical students and caregivers in educational encounters where they take and share perspectives, we need a thorough understanding of the perspective-taking process. To increase our understanding of this interactive process, we studied both how students take perspective, how caregivers share perspective, and connections between both. Our research questions are:

1. Which perspective-taking strategies do medical students use and which facilitators and constraints do they perceive to take a caregiver’s perspective in an educational context?
2. Which strategies do caregivers use to share their perspective with students in an educational context?
3. How do students’ perspective-taking strategies relate to caregivers’ perspective-sharing strategies?

## Methods

We undertook a retrospective qualitative study to analyze student-reflections on perspective-taking and caregiver-interviews on perspective-sharing in an educational context. Ethical approval of this study was provided by the Dutch Association for Medical Education (NVMO, NERB file number: 2020.4.7).

### Context

The context of this study was a mandatory lesson, named Parental Advisory, for fourth year students within the curriculum of the University Medical Center Utrecht, a Dutch medical school. It took place during a 6-week preparatory course preceding an integrated clerkship combining pediatrics, gynecology, and clinical genetics. From March 2020 until December 2021 this lesson was provided online due to the COVID pandemic. The goal of this lesson was for students to obtain insight into caregivers’ perspectives. The lesson, lasting 75 minutes, was provided by two caregivers (parents) of children who had needed extensive medical care (P1 and P2), and one facilitating medical teacher (researcher CE).

The online lesson consisted of five parts. First, to prepare for the lesson, students watched a video in which P1 told her story about the illness of and care for her child. Second, students reflected on the story of P1 through group discussion. This group discussion was guided by P2. P2 asked questions and reflected on students’ answers using their own experiences as a parent. Third, students were divided into break-out rooms of 4–6 students to prepare questions for P1. Fourth, P1 entered the digital classroom and students asked their questions. Lastly, after the lesson students undertook an individual assignment where they audio recorded their reflections on how they took perspective and what they learned from taking perspective. See Appendix A for the assignment. Students submitted their reflection assignment within a week after the lesson. The assignment was mandatory, and ungraded.

### Participants

The two caregivers (P1 and P2) who provided the online lesson had several years of experience in teaching students. In total 23/27 fourth-year medical students, who attended this lesson in July 2020, agreed to participate.

### Data collection

We collected students’ reflective assignments. Students were encouraged to hand them in by means of audio-recording, this facilitates participants to express themselves, say more, and prevent the tendency to create edited or polished/desirable answers as written documents might encourage [39]. In this way, we collected rich and authentic data from a large group of students in an efficient way in close proximity to the actual educational encounter. Five out of 23 participating students submitted written reflections.

The assignment’s reflective questions were based on a study by Gehlbach and Brinkworth on perspective-taking strategies of high-school students and adults from various professions [20]. To test the comprehensibility and interpretation of the assignment, it was first piloted with two medical students who had already completed the lesson. This resulted in minor adjustments to the instructional text, but not to the assignment questions. Students’ submitted recordings lasted median 3.5 minutes.

Caregivers were interviewed individually. The semi-structured interviews were conducted by researcher CE, who was also involved as a facilitating teacher of the lesson. The interview guide consisted of questions on how caregivers tried to share their perspective with students, see Appendix B. The semi-structured interviews were conducted and recorded via online video-calls using Microsoft Teams and lasted approximately 60 minutes.

The student reflections and caregiver interviews were transcribed verbatim.

### Data analysis

Data were analyzed using NVivo 12 (QSR International, Burlington, Massachusetts). Data-analysis comprised 5 steps (see Appendix C for a more elaborate description).First, for the student reflections we performed a template analysis to identify perspective-taking strategies, facilitators, and constraints [40,41]. We chose this method because it allowed for building on previous perspective-taking literature through deduction and left room for inductively developing new codes. A strategy was defined as an activity (either cognitive or non-cognitive) aimed at understanding a caregiver’s perspective. A facilitator or constraint was defined as an aspect that made perspective-taking easier or more difficult. CE and LB iteratively applied an *a priori* template to the first eight transcripts, whilst refining and adding codes. The perspective-taking strategies in our data fitted the strategies reported by Gehlbach and Brinkworth, therefore we adopted their taxonomy to deductively code the perspective-taking strategies [20]. Facilitators and constraints were both deductively and inductively derived. These codes were organized into summary themes by CE and LB, in consultation with RK. The final coding template was applied to the full data set by CE or LB.

Second, we performed a within-case analysis to identify relations between perspective-taking strategies [42]. Text units containing more than one strategy were analyzed to identify if and how strategies were related to each other.

Third, we performed within-case analyses to identify relations between students’ specific perspective-taking strategies and their perceived facilitators/constraints [42]. CE identified relations by analyzing signal words indicating a relation (for instance: then, so, because). Text units showing these relations were checked by and discussed with RK until consensus was reached.

Fourth, we performed a thematic analysis on caregiver data to identify strategies for sharing their perspective [43]. A strategy was defined as an activity (either cognitive or non-cognitive) aimed at sharing perspective. CE inductively and iteratively identified and (re)defined codes, structured these into summary themes, and discussed findings with RK. The final coding template was discussed with the entire research team and consensus was reached on the interpretation of the data. A member check on the perspective-sharing strategies was performed with P1 and P2, which resulted in no changes.

Lastly, we performed a cross-case analysis to explore if and how caregivers’ perspective-sharing strategies related to students’ perspective-taking [42]. CE and RK individually identified indications for associations that indicated connections or explicit disconnections between caregivers’ strategies and students’ facilitators or constraints by comparing codebook descriptions. Then, CE went back to the data to verify whether these hypothetical associations were supported by text units that were coded as a perspective-taking or perspective-sharing strategy. Associations that were supported by the data were checked by and discussed with RK, after which consensus was reached. Final interpretations were discussed with the entire research team.

### Reflexivity

The research team’s different backgrounds provided multiple perspectives on the data. CE and JF are physician-educators focused on patient involvement in education. RK and MS are educational researchers. LB was a 6th-year medical student at the time of this research. Some team members had active teaching roles in the educational context of this study, which might have impacted the collection of data and interpretations of the findings. CE and JF were both involved in developing the lesson. Moreover, CE was involved as the facilitating teacher during the lessons. CE was purposely selected to conduct the interviews with the caregivers, since she knew the educational context and caregivers well. We assumed this would enable CE to ask follow-up questions that would lead to more depth in the interviews, and caregivers would dare to be more open since there was already a trust bond. However, this could have also resulted in aspects remaining unmentioned since these were self-evident for both CE and the caregivers. To enhance reflexivity, biweekly researcher meetings were held during data collection and analysis, where interpretation of the data and underlying assumptions were discussed.

## Results

We found that both students and caregivers used multiple strategies in the process of taking and sharing perspective in the educational context. Students reported various facilitators and constraints for taking the perspective of caregivers. Some of these facilitators were interpreted as connected to caregivers’ perspective-sharing strategies. We first describe the strategies students used to take perspective, what facilitators and constraints they perceived in taking perspective, and how these related to their specific perspective-taking strategies. Then, we describe what strategies caregivers used to share their perspective. Lastly, we describe how caregivers’ perspective-sharing strategies connected to students’ perspective-taking.

### Students’ perspective-taking strategies

We identified eight perspective-taking strategies: five inferential strategies, where students made inferences from available information and three cultivation strategies, where students attempted to elicit more information about the caregiver. Table 1 describes the strategies, and below we illustrate our findings with quotes.

**Table 1:** Medical students’ strategies to take a caregiver’s perspective*.


STRATEGY	DEFINITION: students…

**Inferential strategy**	**use existing information to try to make inferences about the caregiver**

Analogy (A)	… recall a different situation from their own experience or experiences of others that is presumed to parallel the caregiver’s situation

Compare and contrast (C&C)	… use comparisons to identify differences and/or similarities that will aid in understanding the caregiver’s thoughts/feelings

Consider present context (CC)	… evaluate the present context or the situational factors the caregiver is experiencing

Projection, anchoring & adjusting (P)	… imagine themselves in the caregiver’s situation and may adjust for differences between themselves and the caregiver (i.e. students put themselves in the caregiver’s shoes)

Stereotyping (S)	… use generalized schemas to infer caregiver’s thoughts and feelings in a particular situation

**Cultivation strategy**	**engage in regulatory or active behaviors to try to gather more information about the caregiver**

Attention regulation (Ar)	… regulate their attention (for instance by active listening) to maximize communication

Information extraction (Ie)	… elicit more information from the caregiver about the caregiver’s thoughts/feelings

Open-mindedness (O)	… deliberately withhold judgments to remain open or receptive to new ideas, hypotheses, or arguments relating to the caregiver’s perspective.


*Definitions of perspective-taking strategies are largely derived from Gehlbach’s taxonomy of social perspective-taking strategies. [20].

Students used multiple strategies to take the caregiver’s perspective. For instance, a student combined the strategies Attention regulation (Ar), Projection (P), and Analogy (A):

[I adopted the caregiver’s perspective] especially by listening carefully to her story first.(Ar) Subsequently, by putting myself in [the caregiver’s] shoes, by really imagining: how would I feel and how would it be for me.(P) … but what also helped me is thinking back to stories of others who have been in similar but less severe situations. By then thinking back: what was it like for them?(A) – St16

Some strategies were not only used in addition to others, but also supported other strategies. For example, students described using a Stereotype (S) or an Analogy (A) to be better able to Project (P) themselves in the caregiver’s shoes. Moreover, students described Considering the context (CC) that the caregiver was in to be better able to Project (P) themselves in the caregiver’s situation:

‘The accounts of her experiences in the intensive care unit: I’ve personally spent a lot of time there. So, then I try to picture the situation, with all the noise and activity in the ICU.(CC) That moment she described at the very beginning at the radiologist, that she felt pressed against the wall by all the [people in] white coats there. (CC) I can really imagine what that would look like. Then, I construct my own feelings: what would I personally feel like right at that moment?(P)’ -St8

Furthermore, students described Projecting (P) themselves in the caregiver’s situation and using this projection to Compare and Contrast (C&C) between themselves and the caregiver. “I tried to imagine what it would have been like to find myself in such a situation(P), but I don’t think you can give a realistic answer to that question. However, I think I probably would have acted completely different than [the caregiver]. (C&C)”– St3

Lastly, students used an Analogy (A), which could either be a personal experience or experiences of others, which they then Compared (C&C) to the caregiver’s situation, feelings, or thoughts.

‘In an attempt to understand [the caregiver’s] thoughts and feelings, I recalled my experiences of the past year. I have spent the past year working in the Pediatric Oncology department in [another country]. Although that’s obviously a completely different setting and a totally different hospital, you’re dealing with roughly the same situation. … I saw a lot of desperation there, a lot of love, sadness and heartbreak. … You know those mothers are getting the most out every day. (A) I get the idea that that’s what [the caregiver] did as well: make the most of every day she and her child got to spend together.(C&C) – St11

### Students’ perceived facilitators and constraints for taking perspective

We identified seven factors that students perceived as a facilitator or constraint for adopting perspective-taking strategies. We organized these into contextual, individual or caregiver-related factors, See Table 2.

**Table 2:** Medical students’ perceived facilitators and constraints to taking a caregiver’s perspective.


FACILITATORS AND CONSTRAINTS	DESCRIPTION: The student describes that … acted as a facilitator or constraint

**Context**	

Room for interaction^1^	the possibility to interact with the caregivers and ask questions during the lesson

**Individual (perspective-taker)**	

Familiarity with the physical scenery^1^	knowledge of the physical environment where the story takes place (e.g. the intensive care)

Identification with the caregiver^3^	(not) being able to identify with the caregiver by (not) recognizing similarities with the caregiver

Having similar experiences^3^	(not) having experienced similar situations themselves, e.g. being a patient or having an ill family member, or having experiences with other patients in similar situations.

Life-experience^2^	having little life experience

**Target (perspective-sharer)**	

Decipherability^1^	the caregiver being open

Personal detailed narrative^1^	the caregiver bringing the story from a personal perspective, as a narrative with many details


^1^Only mentioned by students as facilitator, ^2^Only mentioned by students as a constraint, ^3^Mentioned by students as facilitator and constraint.

**Figure 1:**
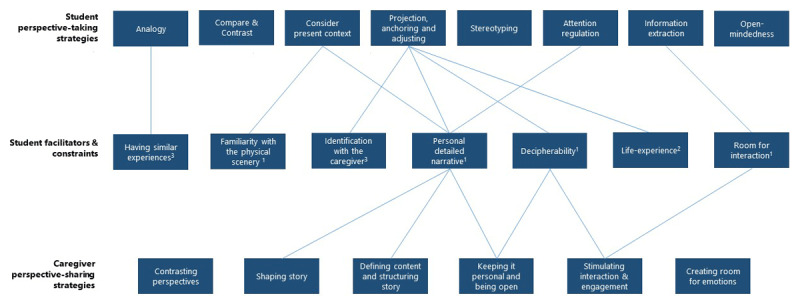
**Overview of perspective-sharing strategies, perspective-taking strategies, and facilitators/constraints for taking perspective.** This figure provides an overview of the results by illustrating students’ perspective-taking strategies, students’ perceived facilitators and constraints for taking perspective, and caregivers’ perspective-sharing strategies. It illustrates how four caregiver’s perspective-sharing strategies were recognized by students as facilitators for taking perspective. Moreover, it illustrates how students’ perceived facilitators and constraints were connected to specific student strategies for taking perspective. (1) Only mentioned by students as facilitator. (2) Only mentioned by students as constraint. (3) Mentioned as both facilitator and constraint.

Students related these facilitators and constraints to specific perspective-taking strategies as depicted in Figure 1. We illustrate this by providing three examples of how strategies were facilitated or constrained.

First, the strategy Analogy was facilitated by Having similar experiences.

“You see, around 10 years ago my father died of an illness, and like in [the caregiver’s] story it happened very suddenly. … I could really relate to her story: doctors who were inconsistent in their communications, nurses who weren’t aware of the situation, doctors who don’t consider our personal circumstances, diagnostic error, and so on and so forth. All that made it very easy for me to understand her feelings.’ – St1

Conversely, not having similar experiences constrained the strategy Analogy. Students who perceived this constraint resolved this by adopting other strategies like Attention regulation, Considering the context, Stereotyping, and Projection, anchoring & adjusting.

Second, regarding the strategy Projecting, anchoring & adjusting, students could feel constrained in projecting themselves in the caregiver’s situation because they could not Identify with the caregiver. Often, they resolved this by adopting other strategies like Attention regulation, or they adjusted their projection:

‘I can’t imagine what it would be like to have a child who’s seriously ill, as I don’t have any children, ill or otherwise. I actually tried to imagine another family member of mine going through a similar experience, and how I would be just as emotionally involved. That did help me to understand her process a little better.’ – St13

Third, the Room for interaction and possibility to ask the caregiver questions acted as a facilitator for the strategy Information extraction. Notably, students who did not ask questions themselves, also benefitted from other students extracting information by asking questions:

‘I also thought it was great that we were given the opportunity to ask additional questions. I think students asked quite a few critical questions, but that this gave us an even better idea not only of [the caregiver’s] situation, but also of [her] attitude to life, [her] partner, the little brother, as well as the communication with the doctors.’ – St14

### Caregivers’ perspective-sharing strategies

We identified six strategies that caregivers used to share their perspective. We structured these strategies in two categories: creating the learning environment and presenting the story, see Table 3. For example, to create the learning environment, parents tried to Create room for emotions: “I also usually point it out: you can tell by looking at me that it does something to me. And I actually kind of do that to put them at ease. That they don’t think oh dear, how awkward, he has to tell his story and is already finding it very difficult.” – P2

**Table 3:** Caregiver’s strategies to share their perspective with medical students.


STRATEGY	DEFINITION: the caregiver describes…

**Creating a learning environment**	strategies to create a trusting learning environment

Creating room for emotions	…creating room for emotions during the encounter, by creating time for emotions and addressing own or students’ emotions

Stimulating interaction & engagement	… stimulating interaction with and between students and stimulating engagement. For instance, by explicitly mentioning that interaction is needed, by inviting students to ask questions and respond to each other, by instructing students to put on their cameras to increase non-verbal interaction

**Presenting story**	strategies to present the story

Contrasting perspectives	…contrasting different perspectives: contrasting between the perspectives of a doctor and a caregiver, or contrasting the perspectives of the two caregivers

Keeping it personal & being open	…being open, being themself, and sharing the story from a personal point of view

Defining content & structuring the story	…structuring the story and, based on students’ interaction during the lesson and on their preparatory written reflections, deliberately defining which elements of their story to present and discuss

Shaping story	…formulating the story in a way that makes it more appealing, powerful or impressive, to improve their storytelling


Moreover, to present their story, parents Defined the content and structured their story by using students’ preparatory reflections: I choose which elements of my story I tell based on those reflections…. you know if you already find a certain theme from the reflections of the students, you can tell a mini-story around that theme, that you can give more examples. –P1

More illustrative quotes of parents’ perspective-sharing strategies are reported in the next section of the results on connections.

One strategy could not be fully executed, namely Creating room for emotions. Caregivers attempted to create room for emotions during the encounter, yet they also addressed the desire to create room for emotions after the encounter. Caregivers mentioned that during physical classes, they usually stayed in the classroom after the lesson was finished to have informal conversations with students who appeared emotional or approached them to have a chat. However, during this online course, students left the digital classroom immediately after the lesson ended. As a result, this strategy could not be fully executed.

### Connections between students’ perspective-taking and caregivers’ perspective-sharing

We explored how caregivers’ perspective-sharing related to students’ perspective-taking by studying the connections between caregivers’ perspective-sharing strategies and students’ facilitators and constraints. Four caregivers’ perspective-sharing strategies were interpreted to be connected with students’ context- and caregiver-related facilitators/constraints, as depicted in Figure 1. We illustrate these associations by providing examples in the section below, see Appendix D for additional illustrative quotes. We did not find instances of explicit disconnections.

Caregivers mentioned that they shared their story from a personal point of view (Keeping it personal and being open) to support students’ perspective-taking. “I think telling the story as I felt it and experienced it, is the quickest way to make someone see the situation through my eyes.” – P2

In parallel, students mentioned that caregivers shared their story from a personal perspective (Personal detailed narrative), which facilitated them in Projecting themselves in the caregiver’s situation (Projection, anchoring and adjusting).

‘[The caregiver] shares her own story based on her experiences, without adding any other information. During the lecture, for example, we don’t learn about how certain medical decisions were made, and that’s because [the caregiver] does not know this herself and tells it from the perspective of her personal experience. That made it a little easier for me to imagine what it’s like to go through such a care trajectory’ -St2

In addition, caregivers deliberately structured and shaped their story for it to become a comprehensive and appealing narrative (Defining content and structuring the story; Shaping story). “Of course, you’re telling a story from start to finish and … by simply giving examples and involving [the students] in the story and possibly embellishing it a little, you know. I think you do have a tendency to exaggerate or downplay certain things, or simply spice them up a little.” – P1

Correspondingly, students mentioned that the way caregivers told their story, as a Personal and detailed narrative, acted as a facilitator for taking perspective. “Maybe it was the way of telling the story, like a narrative, it got her perspective across very clearly.” – St8

Lastly, caregivers tried to stimulate interaction (Stimulate interaction and engagement) and be open.

‘… and stating that no question is off limits or too personal, and you hope that, in a relatively short space of time, we don’t have hours of course, you can still manage to establish a sense of trust within the group; that no question is off limits and that you explain to them that they’re perfectly within their rights to ask questions’-P2

Accordingly, students mentioned that the caregivers being open to all questions (Decipherability) and having the possibility to ask questions (Room for interaction) helped them in taking their perspective. “What helped me was [the caregivers’] openness, also in terms of the questions people could ask during the session. That helped me imagine what it would be like” – St2

## Discussion

Patients are increasingly involved in medical education to share their perspectives. However, little is known about how students take a patient’s perspective and how patients share their perspective during educational encounters. In this study, we used a perspective-taking framework developed in social psychology, to explore the process of perspective-taking and –sharing between medical students and caregivers in an online educational lesson. Students used multiple strategies in support of each other to take a caregiver’s perspective. In concordance with perspective-taking being an interpersonal- and context-dependent process, students perceived individual-, caregiver-, and context-related facilitators and constraints for adopting perspective-taking strategies [24,44,45]. Caregivers supported students’ perspective-taking by adopting multiple strategies to share their story and create a trusting learning environment. By studying both students and caregivers we were able to explore connections between perspective-taking and perspective-sharing strategies.

The findings add to the literature in two ways. First, they show that the taxonomy of perspective-taking strategies described by Gehlbach and Brinkworth also applies to a medical education context where students attempt to take the perspective of a caregiver [20]. Moreover, the study adds to this line of work by illustrating *how* strategies can be used in support of each other. For instance, the strategy Analogy could facilitate the strategy Projection: students could use an analogy to be better able to project themselves in the caregiver’s shoes. Currently, perspective-taking studies in the medical context are mainly built on the work of Batson, which differentiates between two strategies: imagining-other and imagining-self [17,18,22]. Yet, in line with observational studies in other contexts, this study shows that in the medical education context the palette of strategies is more diverse and strategies can be interrelated [21,23,26]. Future research can build on our findings to provide further insight into the outcomes of adopting combinations of perspective-taking strategies, such as patient satisfaction or perspective accuracy. After all, enacting perspective-taking strategies does not automatically mean being accurate in taking perspective [19]. Valuable work has already been done on outcomes of individual perspective-taking strategies [17,22,25,46]. Future studies can explore what kind of initiatives to enhance patient-centeredness in medical education (e.g., interviewing, home visiting, filming daily life, or following a daily regime) support what kind of perspective-taking strategies.

Second, we contribute to the perspective-taking literature by combining data of the perspective-taker with the perspective-sharer. Since perspective-taking is an interpersonal process, the interaction between the perspective-taker and perspective-sharer shapes the process. In this study we illustrated how perspective-taking and perspective-sharing can be studied as interactive processes [24,27]. Adopting this approach revealed that some perspective-sharing strategies of caregivers are recognized by students as facilitators for taking perspective, which we interpreted as connections. Yet, this was not the case for all perspective- sharing strategies. For instance, caregivers deliberately tried to create room for emotions, yet students did not mention their emotions. Even though regulating emotions, meaning regulating their own or the perspective-sharer’s emotions, is a strategy described in Gehlbach’s taxonomy of perspective-taking strategies [20], and has also been described in the medical context before [47]. Possibly students were unaware of regulating emotions, they did not recognize this as a strategy for perspective-taking, or they simply did not mention it in their reflection.

On a practical level, our results can be used to provide students with examples of perspective-taking strategies, discuss how these are facilitated or constraint, and reflect with students on how their personal experiences affect the perspective-taking process. As perspective-taking is a dynamic process, offering students a diverse palette of perspective-taking strategies might help them respond to variations in the context and in the patients they will encounter. For instance, in our study, some students encountered barriers to adopting specific strategies: students reported being different from the patient as a barrier to project themselves in the patient’s shoes. Yet, they persisted in their attempt to take perspective by adopting a different strategy or by adjusting their projection [34]. In order to practice adopting various (combinations of) strategies, we recommend providing students with opportunities to practice perspective-taking in diverse contexts and with various patients. In support of this process, we propose a set of prompts that students can use to practice the perspective-taking strategies (see Appendix E).

The caregivers’ perspective-sharing strategies also offer practical implications for educators. Our findings illustrate that in sharing their perspective, caregivers not only focused on sharing their story, they also aimed to create the right learning environment for students to take their perspective. Their perspective-sharing strategies could be used in teacher training for patients in different kinds of educational settings, like interviewing, shadowing, or filming. However, we should not strive for all patients to participate in a teacher training before they share their story. As such, we cannot expect all patients who share their story to enact all of the strategies mentioned in this article. Nonetheless, some of the strategies can also be enacted by a facilitating educator to support patients in their teaching role. For instance, a facilitating educator can stimulate interaction, create room for emotions and help the patient in structuring the story. Yet, we do stress for educators to be conscious of enacting these strategies in a way which supports patients in sharing their story and not in a way that directs patients what story to tell.

### Limitations

Our results probably under-represent the strategies students used. After all, perspective-taking and -sharing is partly an unconscious process and with our methods we could only measure the strategies students and caregivers were aware of [48,49]. This might be more strongly the case for student strategies as we collected only short audio-recordings from the students versus the elaborate interviews of the caregivers. Moreover, our explorative study design was suitable to identify possible associations between processes of perspective-sharing and perspective-taking. Yet, it was not fit to firmly conclude on the associations we found (or did not find). We suggest using the findings as a base for future studies to further explore how strategies for perspective-taking and -sharing are influenced.

Furthermore, the (combinations of) strategies could be specific for the context of our study. Our study was conducted in an online educational environment, with few distractors and where two caregivers shared their perspective with a whole group of students. These caregivers were experienced in sharing their perspective with medical students, which possibly broadened their palette of perspective-sharing strategies. It is likely that different perspective-taking and perspective-sharing strategies are afforded in other educational settings with patients involved, like home visits where students meet patients and their families in their homes [9]. Moreover, we cannot tell to what extent our findings apply to an authentic clinical setting. The goal of the interaction in this educational setting was set towards students’ learning, in an authentic clinical setting there is also a goal of caring for the patient, which likely affects the perspective-taking and -sharing process. Future studies could study the authentic context in comparison to the educational context. Nonetheless, our findings can be seen as a first step to unravel the process of perspective-taking between students and patients, in diverse contexts, in all its complexity.

Lastly, although perspective-taking has many potential positive outcomes contributing to person-centered care (effective communication [50,51], building relationships [50,52], increased patient satisfaction [18]), imagining how a patient thinks and feels can also lead to negative outcomes like personal distress and feeling troubled, especially when the perspective-taker tries to project themselves in the other’s situation [17,53]. So instead of focusing on positive outcomes of adopting combinations of strategies for perspective-taking or -sharing, future studies should also focus on negative outcomes for students and patients during these encounters.

### Conclusion

This study explored the perspective-sharing and perspective-taking process between caregivers and students in an educational context. By combining data from both the perspective-takers (students) and the perspective-sharers (caregivers), we provide a foundation for further research on how students learn from patients through perspective-taking. The perspective-sharing and -taking strategies described in this study can be used by educators, students, and patients as tools to support initiatives of patient involvement in medical education. This will aid in our goal toward person-centered care, for which perspective-taking is essential.

## Additional File

The additional file for this article can be found as follows:

10.5334/pme.412.s1 Appendices. Appendix A to E.
