# Supplementary material for: Perspective-Taking and Perspective-Sharing in Pediatric Education: Exploring Connections Between Strategies of Medical Students and Patients’ Caregivers
Source: Perspect Med Educ. 2023 Oct 6;2(1):372–84. doi: 10.5334/pme.412 (PMC10558030; doi:10.5334/pme.412)
Supplement: Appendices. — Appendix A to E. [file pme-12-1-412-s1.pdf]

Appendix A Study context and reflective assignment

Figure online Parental Advisory lesson

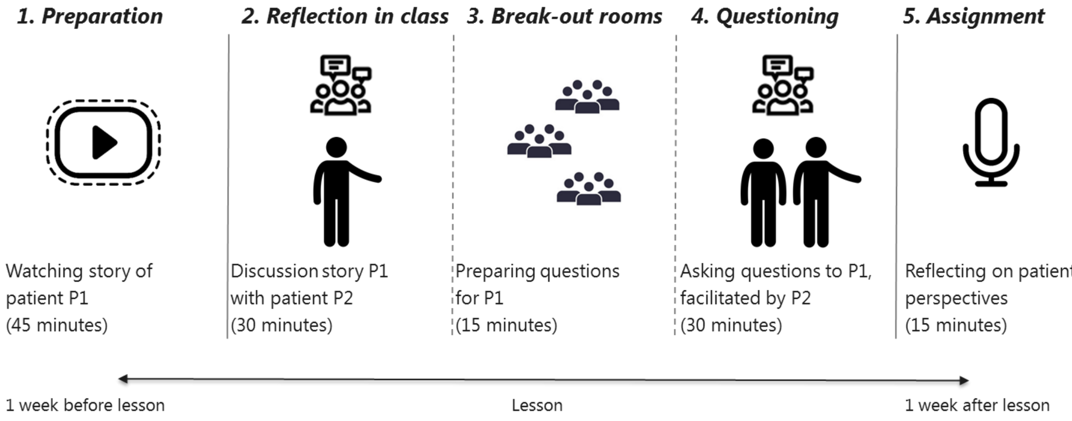

Assignment Parental Advisory lesson

Taking perspective

During the Parental Advisory lesson, we try to develop a better understanding of perspectives of patients' parents. What are their thoughts and feelings with regard to the care for, and care provided to, their child or children?
Prior to attending the lecture, you listened to the story of P and the care of her child. We discussed this story together in the lecture, and there was an opportunity to ask questions to P.

Assignment

You are going to reflect on how you took P's perspective. Think back on P's story, then think back on your own thoughts during the lecture. What did you do, to try to understand P's perspective? What thoughts came up during the lecture when you were trying to understand P's perspective? What helped you in taking his perspective?

Answer the questions below. You don't need to write down your answer, but you audio record your answer on your mobile phone. Just pretend you are sitting across from the teacher and are telling him/her the story. Your recorded message need not be perfect; there are no 'wrong' answers.

Questions

Answer the questions below about how you took P's perspective.

- What thoughts ran through your mind during the lecture when you were trying to understand P's perspective?
- What steps or strategies did you use to figure out P's thoughts and feelings?
- What helped you in taking P's perspective? Why did it help you?
- What have been your takeaways from these classes?

Practical instruction

Record your answers using your mobile phone's voice recorder app, and then upload it to Microsoft Teams.

Appendix B Interview questions patients

<u>GENERAL QUESTIONS</u>

We will start with some general questions

1. **What motivated you to get involved in teaching the Parental Advisory lesson?**
2. **What do you consider the purpose or objective of this lesson?**
    a. Probe - What do you hope students will get out of this lesson?
    b. Probe - What do you *personally* hope to get out of this lesson?

<u>ONLINE PERSPECTIVE SHARING</u>

In class, you share your story and your perspective on illness and healthcare.

3. **How did you try to share your perspective?**
    a. Probe –How did you try to support students in understanding your perspective?
    b. Probe – How do you try to share your story?

4. **What helped you in sharing your perspective?**
    a. Follow-up – How did this help you?
    b. Probe – How did the interaction with students affect the way you share your perspective?

5. **What were barriers for sharing your perspective?**
    a. Probe – For what was this a barrier?
    b. Follow-up –Why did it make this more difficult?

6. **How can you tell your story has resonated with the students?**
    a. Probe – What are indications for this?
    b. Follow-up - Do you feel you succeeded at this?

Appendix C Method – Analyses

We first performed a template analysis to identify students' perspective-taking strategies, facilitators, and constraints. We then used this data for within-case analysis to identify combinations of perspective-taking strategies and combinations between perspective-taking strategies and facilitators/constraints. To identify patients' perspective-sharing strategies we performed thematic analysis. We then combined patient and student data by performing a cross-case analysis identify if and how patient-strategies support students' perspective-taking.

Analysis 1: template analysis student data
Goal: identify student perspective-taking strategies, facilitators and constraints
We performed a template analysis, which is a form of thematic analysis.[1,2] First CE and LB familiarized themselves with the data, by reading the first 8 transcripts. Based on literature, we developed an a priori template containing codes for perspective-taking strategies, facilitating and constraining factors.[3–8] CE and LB iteratively applied this template to the first 8 recordings as follows: CE and LB coded pairs of transcripts separately, discussed their findings, refined and added codes to the codebook, and then used this codebook for following pair of transcripts. Then, in consultation with RK, codes were organized into summary themes and the initial coding template was defined. The facilitating and constraining factors were organized into perspective-sharer, perspective-taker, or context-related factors. The perspective-taking strategies found in our data fitted the perspective-taking strategies reported by Gehlbach *et al*., therefore, we adopted their taxonomy of perspective-taking strategies in our coding template.[4] The coding template was applied to the full data set in two steps. First, CE and LB coded the recordings 9-18 separately. The intercoder agreement for selecting relevant text units was 94% and for codes assigned to the text units 92%. This was considered sufficient.[9] Then, the final template was applied by LB to transcripts 1-8 and by CE to transcripts 19-21.

Analysis 2: within-case analysis perspective-taking strategies
Goal: to identify combinations of perspective-taking strategies.
We performed a within-case analysis with student data to identify combinations of strategies. During template analysis we adopted the rule that the most prominent strategy for taking perspective was coded within text elements, which was in line with the original codebook for perspective-taking strategies by Gehlbach.[4] Yet, text elements often included multiple overlapping or intertwined strategies. Therefore, CE and LB individually selected text units containing more than one strategy and coded the strategies that were used in combination with each other. They discussed their findings until consensus was reached. Then, CE analyzed how the strategies were related to each other by analyzing temporality of the thought process and by analyzing signal words indicating a relation (for instance: then, therefore, as result). Final interpretations were discussed with the entire research team.

Analysis 3: within-case analysis strategies and facilitators & constraints
Goal: to identify associations between students' facilitators & constraints and specific strategies
CE selected text-units of facilitators, constraints and strategies. CE identified associations by analyzing signal words that indicated a link between facilitators/constraints and strategies (for instance: then, therefore, so, because, this helped me with). Text units showing these associations were checked by and discussed with RK, after which consensus was reached. Final interpretations were discussed with the entire research team.

Analysis 4: thematic analysis patient data
Goal: to identify patient perspective-sharing strategies

Patient data was analyzed using an thematic analysis approach.[10] CE familiarized herself with the data by reading the two interview transcripts. She inductively and iteratively identified and (re-)defined codes, structured these into summary themes, and discussed her findings with RK. Ultimately, the codes where then grouped under two broad categories: creating the learning environment and presenting the story. The final coding scheme was discussed with the entire research team and consensus was reached about the interpretation of the data. A member check on the strategies for supporting students' perspective-taking with R. and A. was performed, which resulted in no adjustments.

Analysis 5: cross-case analysis

Goal: to identify if and how patient-strategies support students' perspective-taking

CE and RK individually identified potential associations between patient strategies and students' facilitators/constraints by comparing codebook descriptions for similarities. Then, for each of these associations, CE went back to the data to verify whether these hypothetical associations were supported by text-units. Associations that were supported by the data were checked by and discussed with RK, after which consensus was reached. Final interpretations were discussed with the entire research team.

References

1. Brooks J, King N. Doing Template Analysis: Evaluating an End-of-Life Care Service. *Doing Template Anal Eval an End-of-Life Care Serv*. 2014;(March 2016). doi:10.4135/978144627305013512755
2. Brooks J, McCluskey S, Turley E, King N. The Utility of Template Analysis in Qualitative Psychology Research. *Qual Res Psychol*. 2015;12(2):202-222. doi:10.1080/14780887.2014.955224
3. Gerace A, Day A, Casey S, Mohr P. An Exploratory Investigation of the Process of Perspective Taking in Interpersonal Situations. *J Relationships Res*. 2013;4:1-12. doi:10.1017/jrr.2013.6
4. Gehlbach H, Brinkworth ME. The social perspective taking process: Strategies and sources of evidence in taking another's perspective. *Teach Coll Rec*. 2012;114(1).
5. Gehlbach H. A new perspective on perspective taking: A multidimensional approach to conceptualizing an aptitude. *Educ Psychol Rev*. 2004;16(3):207-234. doi:10.1023/B:EDPR.0000034021.12899.11
6. Gehlbach H. Social perspective taking: A facilitating aptitude for conflict resolution, historical empathy, and social studies achievement. *Theory Res Soc Educ*. 2004;32(1):39-55. doi:10.1080/00933104.2004.10473242
7. Krishnasamy C, Ong SY, Loo ME, Thistlethwaite J. How does medical education affect empathy and compassion in medical students? A meta-ethnography: BEME Guide No. 57. *Med Teach*. 2019;0(0):1-12. doi:10.1080/0142159x.2019.1630731
8. Gerace A, Day A, Casey S, Mohr P. Perspective Taking and Empathy: Does Having Similar Past Experience to Another Person Make It Easier to Take Their Perspective? *J Relationships Res*. 2015;6(September). doi:10.1017/jrr.2015.6
9. Miles MB, Huberman AM. Cross-Case Displays: Ordering and Explaining. In: *Qualitative Data Analyis - An Expended Sourcebook*. SAGE Publications; 1994:207-238.
10. Braun V, Clarke V, Hayfield N, Terry G. Thematic analysis BT - Handbook of research methods in health social sciences. *Springer Nat Singapore Pte Ltd*. Published online 2019:843-860.

Appendix D Examples of alignment between patients' perspective-sharing and students' perspective-taking

Four patient perspective-sharing strategies aligned with students' context- and target-related facilitators/constraints. This table illustrates this alignment by providing exemplar quotes of the combinations.

The perspective-sharing strategy Stimulating interaction and engagement aligned with the perspective-sharing strategies Room for interaction and Decipherability
**Stimulating interaction and engagement**<br>'*and stating that no question is off limits or too personal, and you hope that, in a relatively short space of time, we don't have hours of course, you can still manage to establish a sense of trust within the group; that no question is off limits and that you explain to them that they're perfectly within their rights to ask questions'*-P2<br><br>**- Room for interaction**<br>*"I think because we got the opportunity to talk to him, and ask various questions. That way you can better imagine how they handled things.'-St10*<br><br>**- Decipherability**<br>*"What helped me was [the patients'] openness, also in terms of the questions people could ask during the session"*-St2
The perspective-sharing strategy Defining content and structuring story aligned with the perspective-sharing strategy Personal detailed narrative
**Defining content and structuring story**<br>*"Of course, you're telling a story from start to finish and, by now, you have already filtered out all the examples that are good" – P1*<br><br>**- Personal detailed narrative**<br>"*Because he took us step by step through his story, it became very vivid to me and it made it easier to take his perspective."*-St14
The perspective-sharing strategy Keeping it personal and being open aligned with the perspective-sharing strategies Personal detailed narrative and Decipherability
**Keeping it personal and being open**<br>*"I think telling the story as I felt it and experienced it, is the quickest way to make someone see the situation through my eyes*."-P2<br><br>**- Personal detailed narrative**<br>"*[He] shares his own story based on his experiences, without adding any other information*."-St2<br><br>**- Decipherability**<br>"*It really helped me that he was very open*"-St13
The perspective-sharing strategy Shaping story aligned with the perspective-sharing strategy Personal detailed narrative
**Shaping story**<br>*"by simply giving examples and involving [the students] in the story and possibly embellishing it a little, you know. I think you do have a tendency to exaggerate or downplay certain things, or simply spice them up a little.'* -P1<br><br>**- Personal detailed narrative**<br>"*Maybe it was the way of telling the story, like a narrative, it got his perspective across very clearly."-St8*

Appendix E Prompts for perspective-taking strategies

To take the perspective of a patient you can adopt multiple strategies. These strategies are described in the table below. Since every patient and every situation is unique, different combinations of strategies apply in different situations. We encourage you to practice various combinations of strategies in response to differences in the context and the patients you encounter. You can use the prompts to practice these strategies.

Remember, adopting perspective-taking strategies does not automatically mean you are correct in taking perspective. We encourage you to, when possible, check with the patient if your understanding of her or his perspective is correct.

Strategy	Explanation	Prompt
**Inferential strategy**	**use existing information to try to make inferences about the patient**	
Analogy	… recall a different situation from your own experience, or experiences of others, that is presumed to parallel the patient's situation	• Think about a situation where you, or someone you know, experienced something that is comparable to the patient's situation. How did you, or the person you know, think and feel at that time?
Compare and contrast	… use comparisons to identify differences and/or similarities that will help you in understanding the patient's thoughts, feelings and behavior	• If you were in the situation of the patient, how would you think, feel and/or act?<br>• How would most other people think, feel and/or act in the situation of the patient?<br>• How did other people in the situation of the patient think, feel and/or act?<br>→ How does this compare to how the patient thinks, feels and/or acts? How can similarities or differences be explained?
Consider present context	… evaluate the present context or the situational factors the patient is experiencing	• Think back to the physical context the patient is in: how does the setting look like, what other people are there? How would this affect the thoughts, feelings, and/or behaviour of the patient?
Projection, anchoring & adjusting	… imagine yourself in the patient's situation and adjust for differences between yourself and the patient	• Imagine you are in the patient's situation yourself (put yourself in the patient's shoes). How would you think, feel and act?
Stereotyping	… use generalized schemas to infer patient's thoughts and feelings in a particular situation	• How would people from a similar group, for instance other patients with a chronic condition, typically think and feel in a situation that is similar to the patient's situation?
**Cultivation strategy**	**engage in regulatory or active behaviors to try to gather more information about the patient**	

Attention regulation	… regulate your attention to maximize communication	• Focus your attention to the patient's words, facial expressions and gestures. What is the patient telling or showing you?
Information extraction	… elicit more information from the patient about his thoughts/feelings	• What additional information do you need to have a full picture of the patient's perspective? How can you elicit this information?
Open-mindedness	… deliberately withhold judgments to remain open to new ideas, hypotheses, or arguments relating to the patient's perspective.	• Can there be other reasons for why the patient is responding this way?<br>• What are other thoughts and feelings the patient might experience?
